# Frequency and duration of suffering of cervical cancer patients and caregivers: Results from an international Delphi study

**DOI:** 10.1371/journal.pgph.0001642

**Published:** 2023-03-09

**Authors:** Xiaoxiao Jiang Kwete, Khadidjatou Kane, Yuwei (Alyssa) Liu, Eric L. Krakauer

**Affiliations:** 1 Harvard School of Public Health, Boston, MA, United States of America; 2 Global Health Research and Consulting, Yaozhi, Yangzhou, China; 3 Division of Palliative Care and Geriatric Medicine, Department of Medicine, Massachusetts General Hospital, Harvard Medical School, Boston, MA, United States of America; 4 Division of Palliative Care and Geriatric Medicine, Massachusetts General Hospital, Boston, MA, United States of America; 5 Department of Medicine, Harvard Medical School, Boston, MA, United States of America; 6 Department of Global Health and Social Medicine, Harvard Medical School, Boston, MA, United States of America; 7 Department of Palliative Care, University of Medicine and Pharmacy at Ho Chi Minh, Ho Chi Minh City, Vietnam; University of Washington, UNITED STATES

## Abstract

This paper describes a Delphi process executed between August and September, 2020, to identify types of physical, psychological, social and spiritual suffering and their severity, prevalence and duration associated with cervical cancer to enable estimation of the global and regional palliative care needs of these cervical cancer patients and their family caregivers. Patients were dichotomized into decedents (those who died of cervical cancer in any given year) and non-decedents (those who had cervical cancer in any given year but did not die in that year). A two-round web-based Delphi study was conducted using a panel of 12 experts with first-hand experience taking care of cervical cancer patients and their family caregivers, two from each World Health Organization (WHO) region. We identified thirteen types of physical suffering, six psychological types, three social types and three spiritual types. Frequencies and durations were given for each of the suffering types for a decedent, a non-decedent and a primary family caregiver. Our findings of the types, severity, frequency and duration of suffering associated with cervical cancer should inform global, regional, national and local health care strategic planning so that the health investments can be better aligned with the needs.

## Introduction

Cervical cancer continues to be one of the greatest threats to women’s health and well-being globally, especially in resource constraint areas. In 2020, there are 1.5 million women living with cervical cancer within 5 years of diagnosis, with 600 thousand new cases, 88% of which were found in residents of low- and middle-income countries (LMICs) [1]. Cervical cancer is not only life-threatening, but can also generate severe physical, psychological, social and spiritual suffering for women and caregivers. While five-year survival rate for cervical cancer varies country by country, people in developing countries consistently have lower survival rate than that in developed countries. Less than a quarter of those living in low-income countries survive 5 years after diagnosis [2, 3].

In 2020, the Director General of the World Health Organization (WHO) announced a new initiative—the Global Strategy to Accelerate the Elimination of Cervical Cancer as a Public Health Problem—which was adopted by the 73rd World Health Assembly in August 2020 [4]. One of the three main pillars of this strategy is to make sure women with invasive cervical cancer have access to quality health service, including palliative care that aims to alleviate sufferings of all kinds associated with cervical cancer or its treatment.

To provide optimum palliative care for cervical cancer patients and their caregivers, it is necessary to identify the ways in which patients and caregivers suffer and the severity, prevalence, and duration of each type of suffering. While there are some data on prevalence of symptoms associated with cervical cancer [5–20], most studies are from high-income settings and focus only on physical suffering and/or psychological suffering. There is virtually no data from any setting generalizable data on the duration of symptoms, crucial information to estimate need for and cost of palliative care services for cervical cancer patients.

The Lancet Commission Report on global access to palliative care and pain relief [21], hereafter referred to as the LCR, applied a novel approach to estimate the global need for palliative care, which included 20 different conditions. Experts’ consensus was reached via a Delphi process on frequency and duration of symptoms that can be caused by the 20 conditions, and these data were combined with a global mortality database for the 20 conditions to generate the total number of patients and symptoms-days for each symptom, by condition, and by country. In this dataset, all solid tumors were analyzed together.

To estimate the need for palliative care for cervical cancer patients and their caregivers, a similar method was used. Experts came to consensus on the types, severity, frequency and duration of suffering associated with cervical cancer, and these data were combined with mortality and morbidity data from Institute of Health Metrics and Evaluation (IHME). A global dataset on the annual need of palliative care service for cervical cancer was created, including all four categories of sufferings–physical, psychological, social and spiritual. The results have been published elsewhere [22].

In this paper, we describe in details of the Delphi process conducted to reach the consensus among a group of experts convened. Results from this study can be used to improve both health care service planning and care of individual cervical cancer patients. Experts in other settings can also use this method to determine the needs of patients with any serious illness in any setting.

## Methods

A two-round Delphi method was conducted between August and September 2020 using a panel of 12 experts identified by the authors as having first-hand experience providing health care to patients with advanced cervical cancer and/or with palliative care needs. To be representative, the expert panel consisted of two clinicians from each of the six WHO regions and included 10 medical doctors, one nurse and one social worker. Table 1 summarizes the list of the members of this Delphi panel (see Table 1 for details).

**Table 1:** Delphi panel members.

Country	Occupation	Income Group	WHO Region
India	Physician- palliative care	LM	South-East Asia Region
Senegal	Physician–gynecologic oncology	LM	African Region
Jamaica	Physician–medical and radiation oncology and palliative care	UM	Region of the Americas
Chile	Physician- gynecologic oncology and palliative care	H	Region of the Americas
Botswana	Physician–gynecologic oncology	UM	African Region
Vietnam	Physician–radiation oncology and palliative care	LM	Western Pacific Region
Iran	Nurse	UM	Eastern Mediterranean Region
India	Physician–palliative care	LM	South-East Asia Region
Germany	Physician–psychiatry	H	European Region
Vietnam	Social work	LM	Western Pacific Region
Sudan	Physician–Medical oncology and palliative care	L	Eastern Mediterranean Region
Russia	Physician–palliative care and gynecologic oncology	UM	European Region

A total of 12 individuals were approached, and all of them agreed to participate. Twelve participants (100%) completed round one, and eleven of them (92%) completed round two.

Before the Delphi process, a literature review was conducted by the authors on types and prevalence of suffering experienced by cervical cancer patients and their primary caregiver, and an initial list of major symptoms was created and divided into four domains: physical, psychological, social and spiritual. Physical suffering types included: mild pain and moderate-to-severe pain, dyspnea, fatigue, weakness, nausea and/or vomit, diarrhea, constipation, incontinence of urine, incontinence of stool, malodorous vaginal discharge, bleeding, and dry month. Psychological suffering types included moderate-to-severe anxiety/worry, depressed mood, post-traumatic stress disorders, confusion/delirium, sexual dysfunction, and among primary caregivers only, complicated grief. Social suffering included moderate-to-severe financial difficulties, and feeling stigmatized/discriminated against/socially isolated. Spiritual suffering included moderate to severe loss of meaning in life, loss of faith in/angry with higher power, and higher power causing illness. Following the terminology used by the LCR, we separated the patients into decedents–those who died in any given year, and non-decedents–those who had cervical cancer in any given year but did not die that year. And for the primary caregiver, in addition to complicated grief, we assume that they also experience some of the psychological symptoms, namely, anxiety/worry, depressed mood, and PTSD, as well as all the social and spiritual suffering types that are experienced by patients. A simple mean was used when multiple resources were found for one symptom. No data were found on the duration of any symptom (see Table 2 for details).

**Table 2:** Baseline list of symptoms and references provided for experts.

		Decedents		Non-decedents		Family caregivers	
		Prevalence (%)	References	Prevalence (%)	References	Prevalence (%)	References
**Physical Suffering**	**Pain (mild)**	**96%**	**[5]**	**38%**	**[7]**	NA	
	**Pain (moderate or severe)**	**87%**	**[5, 23]**	**33%**	**[7, 12]**	NA	
	**Dyspnea**	**20%**	**[5]**			NA	
	**Fatigue**	**69%**	**[5]**	**40%**	**[12, 13, 24]**	NA	
	**Weakness**	**56%**	**[5]**	**33%**	**[24]**	NA	
	**Nausea and/or vomiting**	**28%**	**[5, 23]**			NA	
	**Diarrhea**	**8%**	**[9]**	**34%**	**[8, 9]**	NA	
	**Constipation**	**33%**	**[5, 10, 23]**	**38%**	**[9, 10]**	NA	
	**Incontinence of urine**	NA		**47%**	**[7, 9, 10]**	NA	
	**Incontinence of stool**	NA		**35%**	**[7, 10]**	NA	
	**Malodorous vaginal discharge**	**51%**	**[23]**	**38%**	**[25]**	NA	
	**Bleeding**	**66%**	**[17]**	**66%**	**[17]**	NA	
	**Dry Mouth**	**50%**	**[21]**	NA		NA	
**Psychological Suffering**	**Anxiety / worry**	**44%**	**[11]**	**39%**	**[6, 12, 16, 19, 26–28]**		
	**Depressed mood**	**36%**	**[5]**	**29%**	**[6, 12–14, 16, 17, 19, 25–29]**	**19%**	**[30]**
	**Post-Traumatic Stress Disorder**	**29%**	**[6]**	**33%**	**[6, 18]**		
	**Confusion / delirium**	**35%**	**[21]**	NA		NA	
	**Sexual dysfunction**	**78%**	**[31]**	**78%**	**[14, 31]**	NA	
	**Complicated grief**	NA		NA		**7%**	**[20]**
**Social Suffering**	**Stigma and Discrimination**	**24%**	**[25, 32]**	**24%**	**[25, 32]**		
	**Financial Difficulties**	**72%**	**[30, 33, 34]**	**59%**	**[30, 33, 34]**	**32%**	**[30]**
**Spiritual Suffering**	**Loss of meaning**	NA		NA		NA	
	**Anger towards higher power**	NA		NA		NA	
	**Belief higher power causing illness**	NA		NA		NA	

In round one of the Delphi study, results from the literature review were provided to the panelists, including the initial list of symptoms and the prevalence of each symptom summarized from the literature review. Participants were asked to either agree with the reference or provide an alternative frequency of each symptom and fill in a duration of those symptoms based on their experiences caring for cervical cancer patients and their family caregivers. They were also asked whether any other symptoms should be included in the list.

In round two, participants were shown the results from the first round, where a 95% confidence interval was generated without showing any individual’s answers. Symptoms were added to the list based on the number of panelists who proposed it and at the discretion of the research team. The panelists were again asked to estimate in prevalence and duration of each of the symptoms for decedents, non-decedents, primary caregivers. A simple mean was calculated from round two as the consensus.

Data were collected using survey monkey, a third-party online platform, and were collated and analyzed using excel to calculate mean and 95% confidence intervals.

## Results

Results from round 1 of the Delphi process are summarized in Table 3. Only 95% confidence intervals (CIs) are presented. The 95% CI for prevalence is shown as percentages of patients or caregivers who would suffer from each symptom, and for durations, as the number of months each symptom would last per patient or caregiver. Although we only presented 95% confidence intervals, the experts’ estimates converged rather than scattered in most cases, and especially for physical and psychological symptoms. For example, our experts estimated that 95 to 98% of decedents would experience mild pain, while 81 to 88% of decedents would experience moderate to severe pain. And 37 to 47% of non-decedents would experience mild pain, while 25 to 37% of non-decedents would experience moderate to severe pain. Only pain was separated into mild versus moderate to severe levels, while all other symptoms were only at the moderate to severe level. On average, less than one estimate per suffering type fell out of the 95% confidence interval. In round 1, one type of physical suffering and one type of social suffering were added to the list for decedents and non-decedents: leg edema and abandonment by intimate partners, as these were raised by more than half of the participants and deemed important to include by the research team. See Table 3 below for more details.

**Table 3:** 95% confidence interval of the prevalence and duration of each type of suffering for decedents, non-decedents, and family caregivers, from Delphi process step 1.

		Decedents		Non-decedents		Family caregivers	
		Prevalence (%)	Durations (month)	Prevalence (%)	Durations (month)	Prevalence (%)	Durations (month)
**Physical Suffering**	**Pain (mild)**	**(95, 98)**	**(2, 5)**	**(37, 47)**	**(4, 9)**	NA	NA
	**Pain (moderate or severe)**	**(81, 88)**	**(3, 5)**	**(25, 37)**	**(3, 6)**	NA	NA
	**Dyspnea**	**(14, 21)**	**(1, 2)**	**(5, 10)**	**(1, 3)**	NA	NA
	**Fatigue**	**(64, 78)**	**(4, 6)**	**(39, 51)**	**(4, 8)**	NA	NA
	**Weakness**	**(53, 66)**	**(3, 4)**	**(32, 42)**	**(3, 6)**	NA	NA
	**Nausea and/or vomiting**	**(23, 32)**	**(1, 2)**	**(15, 32)**	**(2, 3)**	NA	NA
	**Diarrhea**	**(6, 16)**	**(1, 3)**	**(18, 30)**	**(2, 5)**	NA	NA
	**Constipation**	**(31, 41)**	**(2, 6)**	**(21, 36)**	**(3, 7)**	NA	NA
	**Incontinence of urine**	**(16, 39)**	**(2, 4)**	**(23, 43)**	**(3, 6)**	NA	NA
	**Incontinence of stool**	**(10, 29)**	**(1, 3)**	**(17, 33)**	**(2, 5)**	NA	NA
	**Malodorous vaginal discharge**	**(58, 72)**	**(3, 6)**	**(42, 55)**	**(4, 5)**	NA	NA
	**Bleeding**	**(58, 73)**	**(1, 4)**	**(50, 66)**	**(2, 4)**	NA	NA
	**Dry Mouth**	**(24, 47)**	**(1, 5)**	NA	NA	NA	NA
	**Leg Edema**					NA	NA
**Psychological Suffering**	**Anxiety / worry**	**(53, 69)**	**(4, 6)**	**(43, 55)**	**(6, 10)**	**(39, 59)**	**(5, 9)**
	**Depressed mood**	**(45, 63)**	**(4, 6)**	**(30, 46)**	**(5, 8)**	**(20, 37)**	**(4, 8)**
	**Post-Traumatic Stress Disorder**	**(22, 38)**	**(2, 5)**	**(24, 37)**	**(4, 7)**	**(8, 24)**	**(3, 6)**
	**Confusion / delirium**	**(20, 35)**	**(1, 2)**	NA	NA	NA	NA
	**Sexual dysfunction**	**(82, 89)**	**(5, 6)**	**(68, 87)**	**(7, 11)**		
	**Complicated grief**	NA	NA	NA	NA	**(6, 16)**	**(5, 10)**
**Social Suffering**	**Stigma and Discrimination**	**(33, 61)**	**(3, 5)**	**(28, 46)**	**(6, 10)**	**(6, 28)**	**(3, 7)**
	**Financial Difficulties**	**(70, 91)**	**(5, 6)**	**(59, 80)**	**(6, 10)**	**(47, 76)**	**(7, 10)**
	**Abandonment by intimate partner**					NA	NA
**Spiritual Suffering**	**Loss of meaning**	**(28, 62)**	**(3, 5)**	**(19, 35)**	**(4, 7)**	**(9, 24)**	**(3, 6)**
	**Anger towards higher power**	**(22, 43)**	**(2, 3)**	**(15, 34)**	**(3, 7)**	**(10, 29)**	**(3, 6)**
	**Belief higher power causing illness**	**(15, 41)**	**(2, 4)**	**(12, 35)**	**(3, 7)**	**(7, 32)**	**(3, 6)**

In round 2, 95% confidence intervals, i.e. results in Table 3, were presented to participants, and they were asked to give a new estimate only within the 95% confidence interval, except for the two types of suffering added in the first round, leg edema and abandonment by intimate partner, for which we just took the simple mean from the answers we collected from the second round of the Delphi process. On average, less than one respondent per question responded with a value outside of the 95% confidence interval provided, and those values were removed to calculate the simple mean as the final results. Detailed results were shown in Table 4 below. For certain groups, some types of suffering were shown as “NA” for non-applicable. These data were not collected because the experts regard these suffering types to be either of negligible prevalence or irrelevant.

**Table 4:** The final consensus on the prevalence and duration of each type of suffering for decedents, non-decedents, and family caregivers, from Delphi process round two.

		Decedents		Non-decedents		Family caregivers	
		Prevalence (%)	Duration (months)	Prevalence (%)	Duration (months)	Prevalence (%)	Duration (months)
**Physical Suffering**	**Pain (mild)**	**96**	**5**	**42**	**7**	NA	NA
	**Pain (moderate or severe)**	**84**	**4**	**30**	**4**	NA	NA
	**Dyspnea**	**16**	**1**	**6**	**1**	NA	NA
	**Fatigue**	**71**	**5**	**44**	**6**	NA	NA
	**Weakness**	**58**	**4**	**37**	**4**	NA	NA
	**Nausea and/or vomiting**	**26**	**1**	**20**	**2**	NA	NA
	**Diarrhea**	**11**	**1**	**20**	**3**	NA	NA
	**Constipation**	**37**	**4**	**28**	**4**	NA	NA
	**Incontinence of urine**	**23**	**2**	**28**	**4**	NA	NA
	**Incontinence of stool**	**17**	**2**	**20**	**3**	NA	NA
	**Malodorous vaginal discharge**	**66**	**5**	**49**	**4**	NA	NA
	**Bleeding**	**61**	**2**	**53**	**3**	NA	NA
	**Dry Mouth**	**31**	**3**	NA	NA	NA	NA
	**Leg Edema**	**39**	**3**	**22**	**5**	NA	NA
**Psychological Suffering**	**Anxiety / worry**	**63**	**5**	**50**	**8**	**51**	**7**
	**Depressed mood**	**52**	**5**	**38**	**6**	**28**	**6**
	**Post-Traumatic Stress Disorder**	**28**	**3**	**27**	**5**	**14**	**3**
	**Confusion / delirium**	**26**	**1**	NA	NA	NA	NA
	**Sexual dysfunction**	**87**	**6**	**83**	**11**	**NA**	**NA**
	**Complicated grief**	NA	NA	NA	NA	**11**	**7**
**Social Suffering**	**Stigma and Discrimination**	**52**	**4**	**39**	**8**	**13**	**4**
	**Financial Difficulties**	**84**	**6**	**74**	**9**	**66**	**9**
	**Abandonment by intimate partner**	**46**	**5**	**41**	**9**	NA	NA
**Spiritual Suffering**	**Loss of meaning**	**49**	**4**	**27**	**5**	**12**	**4**
	**Anger towards higher power**	**31**	**2**	**21**	**5**	**18**	**4**
	**Belief higher power causing illness**	**26**	**3**	**20**	**5**	**17**	**4**

The most prevalent physical suffering type among decedents is pain, with experts estimating that 96% of decedents experience mild pain and 84% experience moderate to severe pain, followed by fatigue (71%) and malodorous vaginal discharge (66%). For non-decedents, although mild pain is estimated at 42%, less prevalent than fatigue (44%) and malodorous vaginal discharge (49%), experts also estimated that 30% of non-decedents experience moderate to severe pain. The top three prevalent psychological suffering types for both decedents and non-decedents are the same: sexual dysfunction (87% for decedents and 83% for non-decedents), followed by anxiety/worry (63% for decedents and 50% for non-decedents), and depressed mood (52% for decedents and 38% for non-decedents). Financial difficulties is the most prevalent type of social suffering, for decedents (84%), non-decedents (74%), and family caregivers (66%). The prevalence of difference types of spiritual suffering are similar, and are in general the highest in decedents (26 to 49%), followed by non-decedents (20 to 27%), and then family caregivers (12 to 18%).

## Discussion

Cervical cancer patients, especially those with invasive disease, commonly experience suffering that is more varied and severe than that of other cancer patients. This is due partly to anatomy–the presence of many nerves and nerve plexuses, the tendency to develop fistulae and infections–and partly to the profound emotional significance of the reproductive organs. Treatment regimens for cervical cancer—surgical, medical and radiological—also can have moderate or severe adverse effects. Cervical cancer commonly results in sexual dysfunction that can lead to social stigmatization, discrimination, self-doubt, and depression. It is important to have a comprehensive understanding of the suffering pattern faced by patients. Our work addresses the lack of data on the types, frequency and duration of the most common and severe types of physical, psychological, social, and spiritual suffering. Our data were generated by an initial literature review followed by a Delphi process with an expert panel of 12 professionals with first-hand experience caring for cervical cancer patients and their families.

Our study also identified the most prevalent types of physical suffering–pain, fatigue, and malodorous vaginal discharge, psychological suffering–sexual dysfunction, anxiety/worry, and depressed mood, and social suffering–financial difficulties, that provides further insights into the experience of cervical cancer patients and their family care givers from professionals around the world. In general, non-decedents have lower prevalence of suffering than decedents but not shorter in duration. Family caregivers suffer the least, but even they are estimated to bear a considerable proportion of psychological, social and spiritual suffering. For example, expert estimate that more than half of family caregivers suffer from anxiety/worry, about two thirds of them experience financial difficulties, and one out of every six or eight of them suffer from either loss of meaning in life, anger towards higher power, or belief that the high power has caused the illness. It is critical to take into account the family caregivers of cervical patients when formulating policy support for this group.

As a typical Delphi process [35], our study brings the limitation of lack of context: a panelist might not have a strong impression of the prevalence of some types of suffering, particularly in radically different contexts. Thus, results of a literature review were provided to help the panelists anchor their thoughts. All of them were encouraged to express any disagreement with the literature review and base their responses to the survey on their professional experience.

How patients suffer from cervical cancer is largely dependent on the stage at diagnosis, quality of treatment and palliative care, their socio-economic status, as well as the political and cultural environment of the societies they are in [36]. Thus, we did see a wide range of answers provided by experts from widely varied settings at the end of round one. Although a simple mean was created as the consensus, we recognize that the results only represent the average suffering pattern of “typical” cervical cancer patients, and variations will be commonly observed.

All Delphi study are limited in that the results come from experts’ opinions. But since little literature exists on the suffering and symptom burden of cervical cancer patients and their family caregivers, especially in low- and middle-income countries, the information from our study is in our view the most reliable currently available. We encourage palliative care researchers and policy makers to form their own expert Delphi panels to generate evidence that is closer to their local context. The process described in this paper could serve as an example of how such studies can be conducted. For care providers and relevant government departments who would like to expand their service provision to cervical cancer patients and their families, or to improve the quality of care that is currently available, results from this study could also serve as a guideline for infrastructure building, capacity strengthening, and medication and medical device procurement, as we provided a detailed list of sufferings faced by their target population.

The result from this Delphi study is helpful in estimating the total need of palliative care for cervical cancers in any given region, country or municipal units within countries. It can help palliative care advocates estimate the total needs for palliative services among their target populations by simply multiplying the total number of patients they serve with the frequency and duration of each of the symptoms. The high frequency and duration of almost all symptoms is an indication that the majority of cervical cancer patients continue to lack access to effective palliative care across the world. While clinical guidelines for palliative care for those patients are widely available, more investment needs to be made to make sure services are in place to implement those guidelines. It is morally imperative to protect women from unnecessary and debilitating suffering. This requires integration of palliative care with cervical cancer prevention, early diagnosis, and treatment, and with primary health care as a necessary step toward achieving universal health coverage [37].

## Supporting information

S1 Data Data-file contains two working sheet. The first sheet contains results from the step 1 Delphi, including de-identified individual responses (row 3 to 14), the upper and lower bound (row 27 and 28), the mean and median (row 35 and 36), the standard deviation and the 95% confidence interval (row 42 to 44), and whether the values provide in Table 2 were out of the 95% confidence intervals. Colors were explained in notes following each step. The second sheet contains results from the step 2 Delphi, including de-identified individual responses (row 3 to 13), the upper and lower bound (row 22 to 25), the reconstructed responses (row 29 to 39), and the final results (row 42, 43, and 47).
(XLSX)
